# Fingertip soft tissue defect caused by periungual warts: A case report

**DOI:** 10.1002/ccr3.7866

**Published:** 2023-09-30

**Authors:** Xiaoqin Wang, Jinhui Xu, Yourang Jiang, Deli Zhang

**Affiliations:** ^1^ Department of Dermatology Chongqing Traditional Chinese Medicine Hospital ChongQing China

**Keywords:** case report, periungual warts, soft tissue defect, superficial x‐ray therapy

## Abstract

Periungual warts are frequently encountered in the field of dermatology. Here, we describe the case of a 69‐year‐old individual who presented with hand warts. The wart growth extended to the finger stump, resulting in a soft tissue defect on the fingertip of the right thumb. A treatment approach involving superficial x‐ray therapy in combination with tretinoin was employed to address this finding. The warts disappeared after completing 26 days of the treatment regimen. Fingertip soft tissue defects due to periungual warts are a rare occurrence in clinical settings. This report serves as the first documented case of such a problem successfully managed with the treatment approach mentioned above.

## Graphical abstract

Fingertip soft tissue defects caused by periungual warts have rarely been seen in clinics. This is the first clinical report documenting a fingertip defect caused by warts, which were effectively treated with minimal side effects.

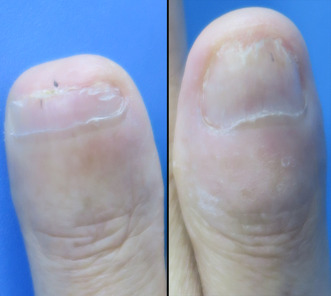

## 1 INTRODUCTION

Periungual warts develop around the nails or nail margins, causing harm to the nail bed and impeding the normal growth of the nail.^1^ Asymptomatic infections with human papillomaviruses (HPVs) occur frequently, and the resulting lesions manifest as smooth, flat, or slightly raised papules, typically with a skin‐colored or pigmented appearance. The majority of these lesions are managed or eliminated by the body's cellular and humoral immune responses.^2^ Consequently, instances of tissue defects caused by warts are exceedingly rare. In this report, we present the first documented case of fingertip damage resulting from periungual warts.

## 2 CASE PRESENTATION

**FIGURE 1:**
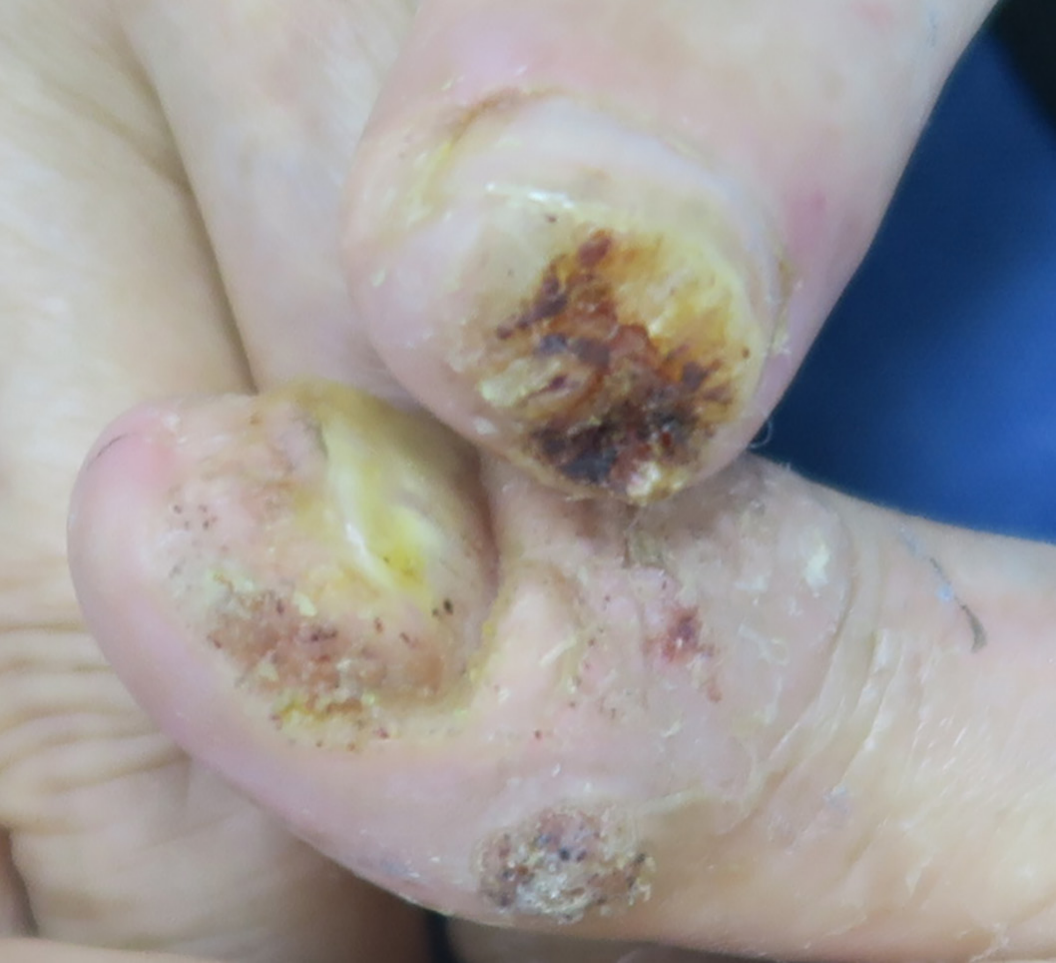
The right thumb's fingertip exhibited a structural defect, with warts extending toward the finger stump.

In September 2021, a 69‐year‐old male patient visited our dermatology department due to hand warts (Figure 1). The patient's diagnosis was based solely on clinical observation, and no additional investigations were conducted. The examination revealed a soft tissue defect on the fingertip of his right thumb, with warts extending to the finger stump. The patient stated that the affected finger was in normal condition before the occurrence of the warts. The thumb on his left hand also displayed periungual warts, leading to nail damage. The patient mentioned having pimples on this finger for over 10 years, which had progressively worsened over the past 4 years. The patient had not received any prior treatment for this condition and had no family history related to it. The patient worked as a caregiver for a colostomy patient and managed excrement from the fistula, which might have caused this condition. The patient did not experience any physical discomfort except for itching. However, these lesions had a psychological impact, causing the patient to feel shame about displaying his hands and fearing that he might infect others. The patient did not report any other diseases.

**FIGURE 2:**
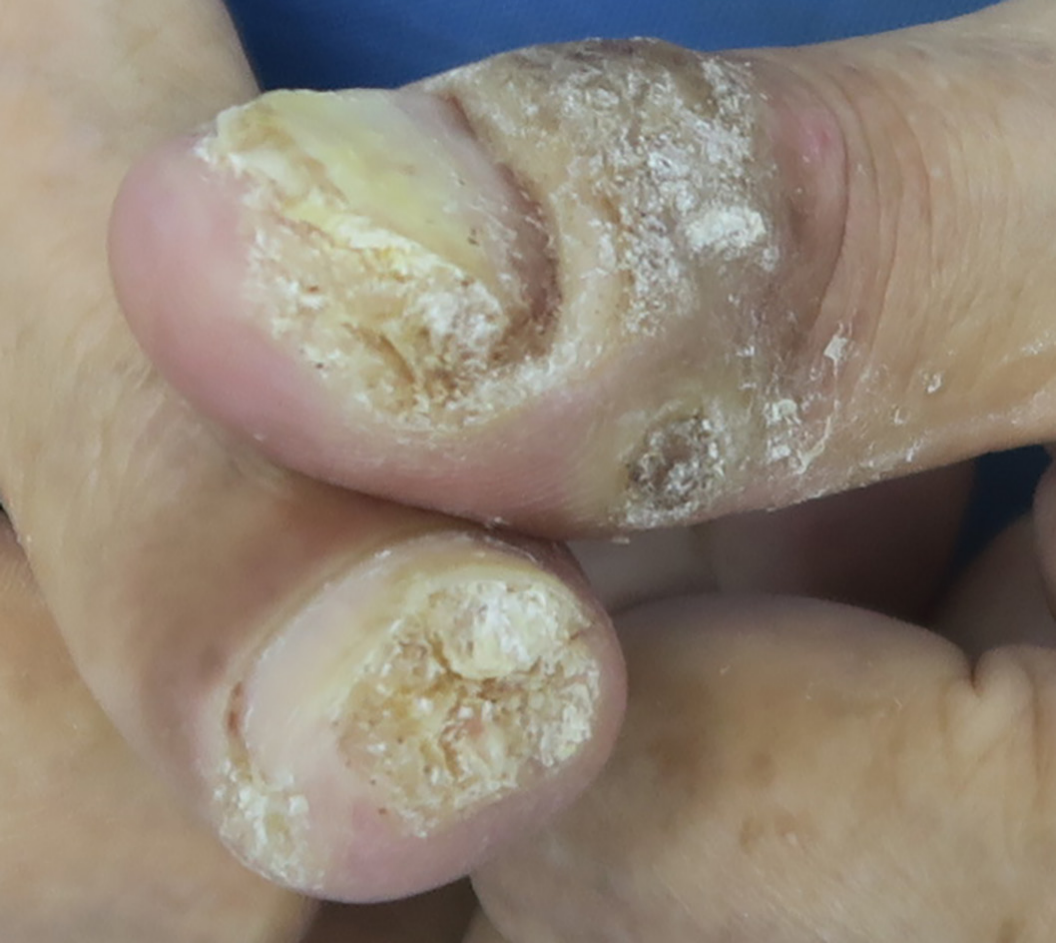
The warts began to shed after three fractions.

**FIGURE 3:**
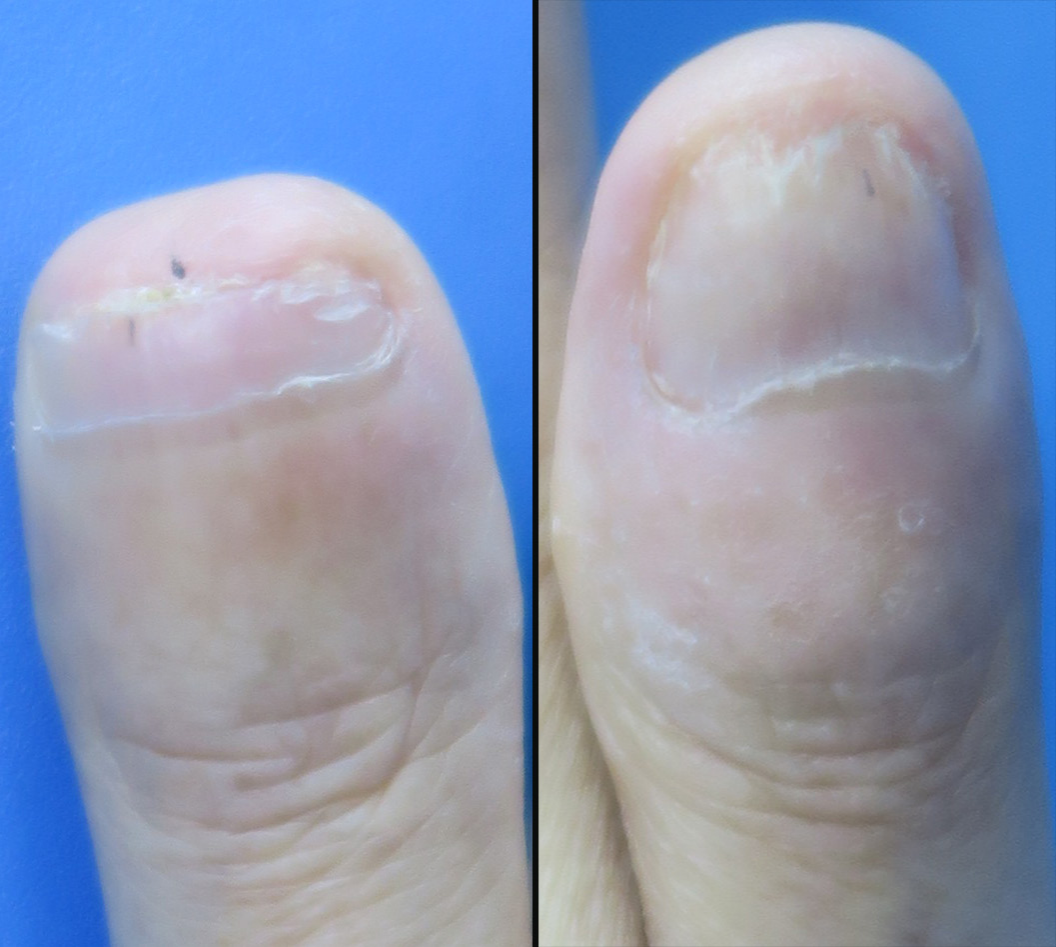
The lesion was completely removed on the right hand after treatment completion.

Our previous study demonstrated that superficial x‐ray therapy (SXRT. SRT‐100, Sensus Healthcare, Boca Raton, FL, USA) in combination with tretinoin proved effective for periungual warts.^3^ Therefore, the patient in the current case study was treated with SXRT in combination with tretinoin. We administered a 5 Gy fraction per week, reaching a total dose of 20 Gy. We also evenly applied 0.1% tretinoin cream (Med‐Xine Pharmaceutical Co. LTD, Sichuan, China) and wrapped the lesion with Saran Wrap for 8 h a day for 20 days, starting from the first fraction of radiotherapy. The patient exhibited good compliance. The warts began to shed after three fractions and disappeared 26 days after finishing the treatment (Figures 2 and 3). The patient experienced mild pruritus. No recurrence was observed during the 1‐year follow‐up period.

## 3 DISCUSSION

HPV is the underlying cause of warts. Once infected, the virus triggers the host cell to undergo division and replicate the viral DNA.^4^ Cellular replication triggered by viral DNA amplification culminates in the development of hyper‐keratinized papules, ultimately resulting in the formation of warts.^5^ However, tissue defects caused by warts are rare. The present study reported a case of an untreated patient with a roughly 4‐mm fingertip tissue defect lesion. These lesions could have been a consequence of a prolonged lack of treatment. However, exposure to alkaline intestinal fluid from his role as a caregiver might have exacerbated the soft tissue damage.

Standard therapy methods for periungual warts include cryotherapy, laser treatment, surgery, interferon, and local drug therapy. However, these approaches often result in severe pain, scar ulcers, and irreversible damage to the nail matrix, leading to nail deformation.^6^, ^7^ Therefore, the commonly used methods for the clinical treatment of warts are limited by their side effects.

Vitamin A is an effective treatment for common warts.^8^ It can interrupt HPV replication and epithelial cell differentiation.^9^ Tretinoin is a generic medication derived from vitamin A, which can alter the abnormal follicular formation caused by the excessive keratinization of epithelial cells.^10^ It can also facilitate cornified cell detachment and increase mitotic activity, thereby increasing the turnover of loosely adherent corneocytes.^10^

Radiotherapy represents a conventional approach to treating warts. The total dosage varies from 1200 r to 3000 r (1 r corresponds approximately to 0.876 cGy), and it can be given in a single application or divided into four fractions.^3^, ^11^ However, the carcinogenic side effects of radiotherapy have nearly halted its utilization for wart treatment. Nonetheless, a previous study demonstrated that superficial radiotherapy was safe when administered at an appropriate dosage.^11^ Our previous study found that the combination of SXRT and tretinoin was highly effective in treating periungual warts while causing only mild side effects.^3^ Considering the increased likelihood of side effects, such as radiation ulcers, with higher dosages, we opted to administer a 5 Gy fraction per week, reaching a total dose of 20 Gy. The patient's warts started to shed after three fractions and were entirely eradicated 26 days after concluding the treatment (Figures 2 and 3). The patient experienced only mild pruritus and no recurrence was detected during the 1‐year follow‐up period.

Based on the currently available information, this is the first clinical report documenting a fingertip defect caused by warts, which were effectively treated with minimal side effects. We recommend the use of SXRT in combination with tretinoin for refractory warts, particularly when conventional treatments are associated with acute pain. However, we acknowledge that our report may have certain biases, as the clinical presentation could have been influenced by contact of the affected areas with alkaline intestinal fluid.

## AUTHOR CONTRIBUTIONS

**Xiaoqin Wang:** Conceptualization. **Jinhui Xu:** Data curation. **Yourang Jiang:** Investigation. **Deli Zhang:** Project administration.

## FUNDING INFORMATION

No funding sources have supported this work.

## CONFLICT OF INTEREST STATEMENT

All authors have contributed to the writing of the manuscript.

## CONSENT

Written informed consent was obtained from the patient to publish this report in accordance with the journal's patient consent policy.

## Data Availability

Data availability is not applicable to this article as no new data were created or analyzed in this study.
